# IQ Motif-Containing G (*Iqcg*) Is Required for Mouse Spermiogenesis

**DOI:** 10.1534/g3.113.009563

**Published:** 2013-12-20

**Authors:** Tanya P. Harris, Kerry J. Schimenti, Robert J. Munroe, John C. Schimenti

**Affiliations:** Department of Biomedical Sciences and the Department of Molecular Biology and Genetics, Cornell University, Ithaca, New York 14853

**Keywords:** forward genetics, reproductive biology, flagellum, spermatogenesis, mouse

## Abstract

Spermiogenesis in mammals is the process by which the newly formed products of meiosis, haploid spermatids, undergo a dramatic morphological transformation from round cells into flagellated spermatozoa. The underlying genetic control of spermiogenesis is complicated and not well-characterized. We have used forward genetic screens in mice to illuminate the mechanisms of spermatozoon development. Here, we report that the oligoasthenoteratospermia in a male-specific infertility mutant (*esgd12d)* is attributable to disruption of a gene called *Iqcg* (IQ motif-containing G). The causality of the mutation was confirmed with a targeted null allele. Loss of *Iqcg* disrupts spermiogenesis such that tail formation either occurs incompletely or breaks apart from the sperm heads. Orthologs are present in diverse species as distant as hemichordates, mollusks, and green algae. Consistent with a conserved role in flagellar formation and/or function, the orthologous *Chlamydomonas* protein is present in that organism’s flagella. Because IQ motif-containing genes typically regulate calmodulin (CaM), which in turn can impact the actin cytoskeleton, these findings suggest a potential role for localized calcium signaling in sperm flagellum morphogenesis.

On completion of meiosis in mammals, the newly formed round haploid spermatids begin a dramatic morphological transformation in which they elongate and form a flagellum and a specialized sperm head. To compact the chromatin into the small head of a mature spermatozoon, the DNA is repackaged by replacing histones with transition proteins and then protamines. This dramatic chromatin remodeling is accompanied by equally remarkable changes in transcriptional and posttranscription gene regulation. The nucleus becomes overlaid by a structure called the acrosome, which is formed from the Golgi apparatus and contains enzymes required for interacting with and fertilizing eggs. The flagellum elongates from the base of the nucleus and contains the motor apparatus, the flagellar axoneme, in its core. To supply the axoneme with ATP, the flagellar midpiece is lined with mitochondria and the flagellar principal piece contains glycolytic enzymes anchored to its fibrous sheath. At the end of spermiogenesis, which lasts approximately 14 days in mice, most of the cytoplasm is sloughed off as a “residual body.”

The genetic control of spermiogenesis in mammals is intricate and poorly understood. Transcription of genes that encode proteins used in spermiogenesis occurs both premeiotically and postmeiotically (Iguchi *et al.* 2006; Gan *et al.* 2013; Elliott 2003). Gene expression profiling studies have estimated that 4% of all mouse genes are spermiogenesis-specific (>900 of all protein-coding genes), and a proteomic analysis found that ∼14% of all proteins in haploid male germ cells are testis-specific (Guo *et al.* 2010). These molecular data underscore the unique developmental complexity of spermiogenesis. Unfortunately, the lack of culture systems to recapitulate spermiogenesis has precluded *in vitro* studies (*i.e.*, use of transfections or siRNA knockdowns), necessitating the use of mouse knockout and conditional mutants to understand the roles of genes expressed in spermatogenesis. Examples of gene mutations causing disruption of spermiogenesis include the transcriptional activator CREM (cAMP response element modulator) (Blendy *et al.* 1996; Nantel *et al.* 1996), the TATA box binding protein (Zhang *et al.* 2001), and the testicular RNA helicase (*Grth*/*Ddx25*), with the latter highlighting the importance of posttranscriptional regulation (Tsai-Morris *et al.* 2004).

These few examples illustrate that genes required for progression from round to elongated spermatids are involved in fundamental but spermatid-adapted cellular processes. Beyond the elongated spermatid stage, there are many mutations affecting spermatozoon morphogenesis and function. The underlying genes can encode structural components of the flagellum, such as the axoneme. According to Mouse Genome Database (Eppig *et al.* 2012), more than 200 genotypes have been reported that cause teratozoospermia, and the culprit genes have not been identified in all cases. Clearly, we are a long way from identifying and understanding the roles of all genes required for normal spermiogenesis and fertility. One useful strategy to identify such genes is forward mutagenesis, in which the mouse genome is randomly mutated to yield phenotypes of interest, followed by identification of the culprit mutations. Previously, we conducted forward genetic screens for mouse infertility mutants, resulting in the isolation of numerous infertile strains with varying phenotypes (Ward *et al.* 2003; Handel *et al.* 2006). Here, we report a male-specific infertility mutant in which the genetic lesion is traced to a gene of previously unknown function called *Iqcg* (IQ motif-containing G). These mice exhibit spermiogenesis defects and reveal the existence of a mechanism of flagellar morphogenesis that is evolutionarily conserved from mammals to unicellular flagellates.

## Materials and Methods

### Positional cloning of *esgd12d*

The isolation of this mutant, previously called 12d and officially called early spermiogenesis defective 12d (*esgd12d*; MGI ID 3050605), by ENU (N-ethyl-N-nitrosourea) mutagenesis was described by Ward *et al.* (2003), who also described rough linkage mapping to Chr 16. For fine mapping, F1s between *esgd12d* (which was induced on the C57BL/6J, or “B6,” version of Chr 16) and C3H or *Mus castaneous* were bred and intercrossed, and recombinant offspring were collected for genotyping with polymorphic microsatellite markers. Males carrying at least one chromosome bearing a B6 allele in the genetically defined *esgd12d* region in *trans* to a recombinant chromosome were fertility tested. Females bearing recombinant chromosomes or males bearing a recombinant chromosome in *trans* to a wild-type (WT) chromosome were crossed to known heterozygotes for *esgd12d* to derive male progeny bearing the recombinant chromosomes in *trans* to the *esgd12d* mutation, followed by fertility testing. Relevant recombinant chromosomes that were genotyped and tested are shown in Supporting Information, Table S1. The mutation was mapped to an interval between *D16Mit58* (32.3 Mb) and a custom primer pair called 12d-4 (33.965 Mb; primer sequences: GATAGAGCACTTGGCCTGGTA and GAAAACCAATGTTTCAGTTTTCA).

### Generation of knockout mice

The targeted ES cell clone EPD0105_3_F01 was acquired from EUCOMM (European Conditional Mouse Mutagenesis program). This was derived from C57BL/6N ES cells and contains an exon-trapping cassette (SA-βgeo–pA) flanked by Flp-recombinase target (FRT) sites within the intron upstream of exon 6 and loxP sites on either side of exon 6 (Figure S1). The targeted line was generated at The Wellcome Trust Sanger Institute and has the official allele name of *Iqcg^tm1a(EUCOMM)Wtsi^*. These ES cells were injected into B6/(Cg)-*Tyr ^c-2J/2J^* albino mice and the resultant male chimera was crossed again to B6/(Cg)-*Tyr ^c-2J/2J^* to achieve germline transmission. Subsequently, this allele was crossed to the Tg(Stra8-cre)1Reb transgenic mouse (obtained from The Jackson Laboratory) (Sadate-Ngatchou *et al.* 2008) to catalyze germline removal of exon 6 while also leaving the gene trap portion intact. We refer to this allele as *Iqcg^KO^*.

### Western analysis

Testicular protein extracts were electrophoresed in 10% SDS/PAGE gels and then electroblotted to Trans-Blot nitrocellulose membranes (BioRad). After blocking with 5% nonfat milk in Tris-buffered saline Tween-20 (TBST), membranes were incubated with rabbit anti-IQCG (1:500; Sigma Prestige Antibodies) or mouse anti-α-tubulin (1:10,000; Jackson ImmunoResearch), followed by HRP-conjugated secondary antibodies. Signal was detected using a Pierce ECL Western blotting substrate detection kit.

### Evolutionary comparisons of proteins

ClustalW2 was used for building an evolutionary tree. The species and accession numbers are as follows: *Chlamydomonas reinhardtii* XP_001689998.1; Hemichordate *Saccoglossus kowalevskii* XP_002738856.1; Lancelet *Branchiostoma floridae* XP_002593396.1; *Ciona intestinalis* XP_002120733.1; mollusk *Crassostrea gigas* ABY27366.1; *Hydra magnipapillata* XP_002154159.1; Frog *Xenopus laevis* GI:76779602; Zebrafish *Danio rerio* XP_002665401.1; Chicken XP_422733.1; Mouse IQCD NP_083684.1; and *Drosophila mojavensis* XP_001999517.1.

## Results

### The *esgd12d* mutation impairs spermiogenesis and causes detachment of sperm heads from tails

**Figure 1:**
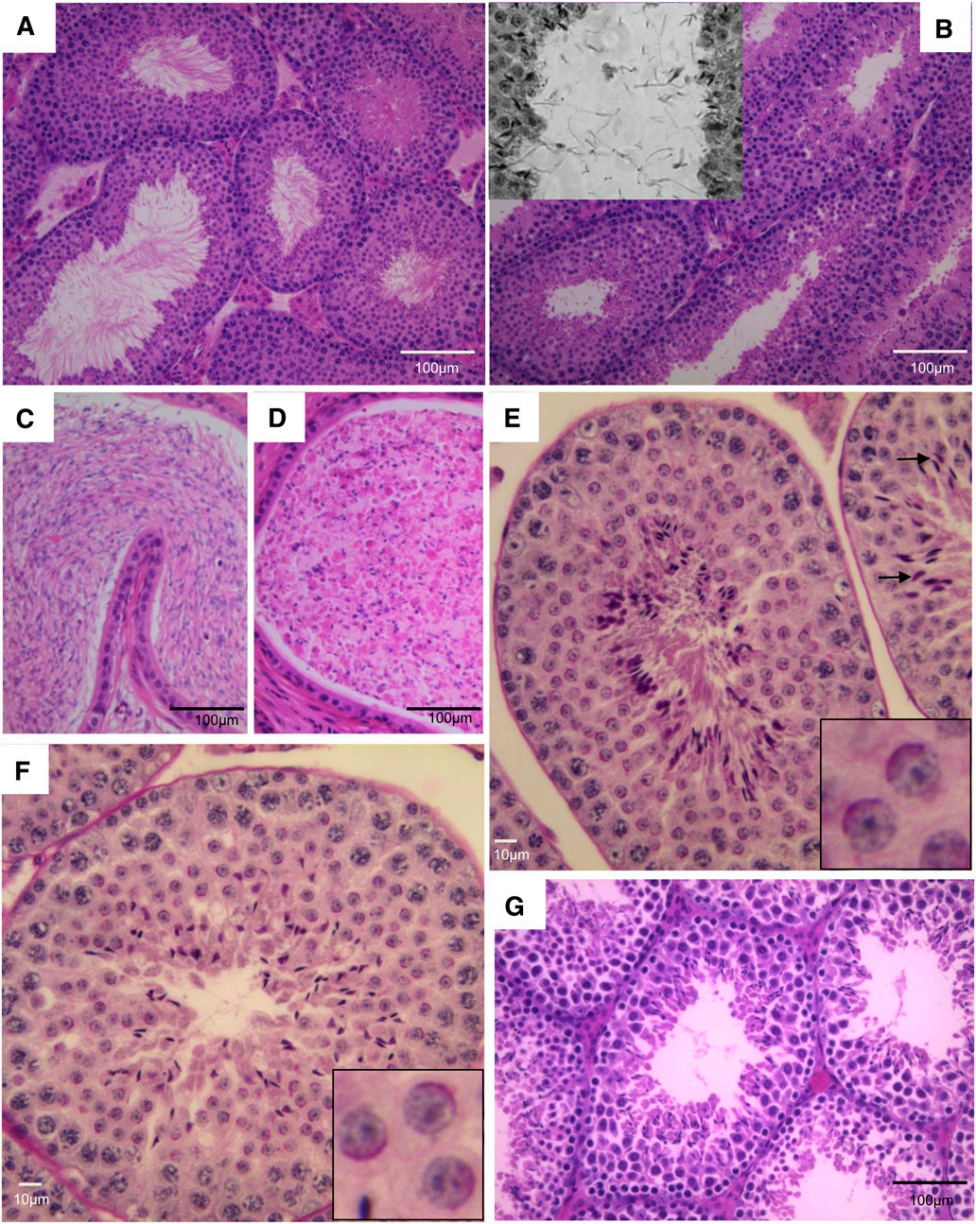
Phenotype of *esgd12d* mutants. (A) Cross-section of a wild-type testis stained with H&E shows seminiferous tubules with numerous spermatozoa, almost ready to spermiate, with their tails filling the lumens. In *esgd12d* mutants (B), most sperm do not develop tails but if they do, the heads break apart from the tails (see inset). (C and D) H&E-stained paraffin sections of epididymides from wild-type (WT) and *esgd12d/esgd12d* mutants, respectively. (E and F) Testis sections from WT and *esgd12d/esgd12d* mutants, respectively, stained with PAS, which binds the acrosome. The magnified insets show that the acrosomal caps are normal in the mutant. (G) H&E section of *Iqcg^Esgd12d/KO^* testis. The phenotype is indistinguishable from *esgd12d/esgd12d* mutants (B).

The early spermiogenesis defective 12d (*esgd12d*) mutation, previously reported as 12d (Ward *et al.* 2003), causes male-specific infertility. Histological sections of WT testes stained with hematoxylin and eosin (H&E) contain seminiferous tubules with elongating spermatids or spermatozoa with tails protruding into the lumen (Figure 1A). Mutant tubules contain postmeiotic cells including elongating spermatids, but most of these cells appeared to lack tails as visualized by a marked deficiency of flagella protruding into the lumens (Figure 1B). Many seminiferous tubule sections also contained sperm tails in the lumen that were unattached to a cell body (Figure 1B, inset). Consistent with these observations, whereas WT cauda epididymides were replete with spermatozoa (Figure 1C), mutant epididymides (Figure 1D) were filled mostly with debris, round cells, and sperm heads. Staining of testis sections with Periodic Acid Schiff (PAS), which highlights the acrosome, revealed that both mutant and WT round spermatids had apparently normal acrosomal cap development (Figure 1, E and F).

**Figure 2:**
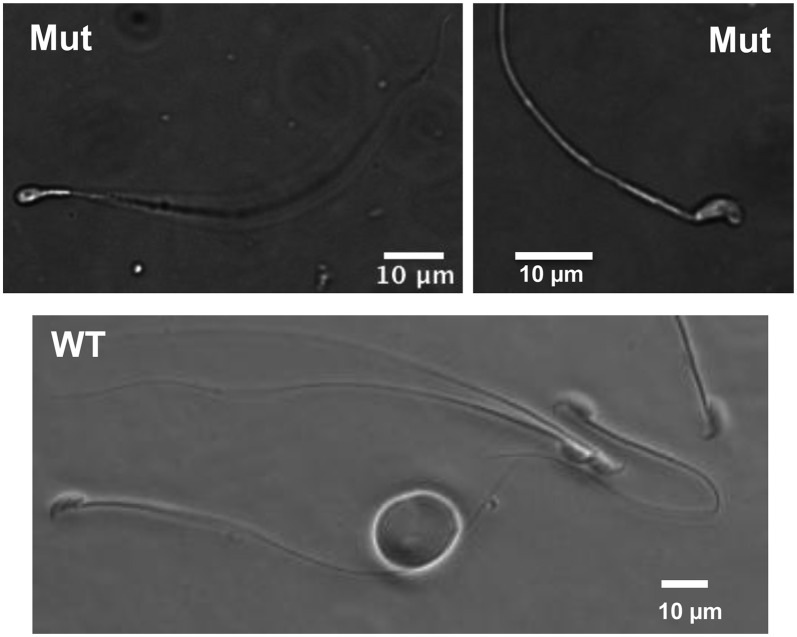
Sperm abnormalities in *Iqcg* mutants. The rare mutant sperm that retained a tail typically had misshapen heads, unlike the typical hook-shape head in wild-type (WT) sperm.

To better characterize the effects of the mutation on sperm maturation, we minced epididymides and allowed sperm to swim into media. Unlike control sperm that are nearly all motile and normal in appearance, mutant sperm with a knockout allele were nonmotile. The lack of mutant sperm motility made precluded accurate quantification, because they could not swim out of epididymis ducts. Overall, >97% of mutant were abnormal morphologically (N = 4; 100 sperm counted per mouse). As predicted from the histology, the most notable abnormality with mutants was the lack of an attached tail to >83% of sperm heads *vs.* <2% in WT (N = 3 for each genotype; 100 sperm counted per male). The remaining mutant sperm (with tails) typically exhibited abnormal heads (Figure 2).

### *Esgd12d* is an allele of *Iqcg*

**Figure 3:**
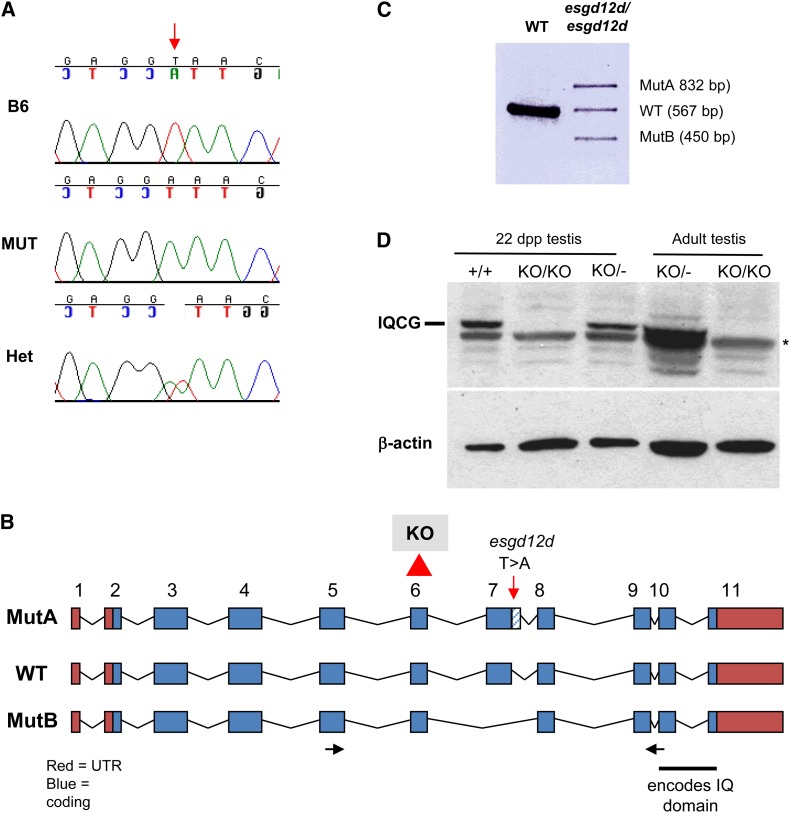
*Iqcg* is mutated in *esgd12d* mice. (A) Sanger sequence traces of *esgd12d* mutation (“MUT”) in the *Iqcg* gene. B6 = C57BL/6J wild-type control. Het = heterozygote for *esgd12d*. (B) Genomic structure of *Iqcg* and mutations. The location of the *esgd12d* point mutation is indicated. This splice site alteration (see text) results in two abnormally spliced isoforms, MutA and MutB, detected by RT-PCR and shown in (C). In the targeted allele, exon 6 is deleted as indicated. (D) Western blot analysis reveals a complete absence of IQCG protein in homozygous mutant testes. The presence of IQCG at 22 days postpartum (dpp) indicates that protein is normally present in round spermatids and possibly earlier cell types.

High-resolution genetic mapping of *esgd12d* (see *Materials and Methods* and Table S1) allowed us to localize the mutant locus to a 1.7-Mb region of Chr 16 containing ∼24 genes. Sequencing of candidates uncovered a mutation (Figure 3A) in the *Iqcg* gene, named as such because it contains a single “IQ” motif that signifies the first two consensus amino acids of the 11 aa consensus motif: [FILV]Qxxx[RK]Gxxx[RK]xx[FILVWY]. Gene expression profiling data indicate that this gene is transcribed predominantly and almost exclusively in testis (Su *et al.* 2004). The *esgd12d* allele contains a T > A change within the consensus splice site at the end of *Iqcg* exon 7 (AG/GT to AG/GA, with “/” representing the termination of the exon) (Figure 3B). Sequencing of RT-PCR products from the mutant revealed that this causes two types of abnormal products: the usage of a cryptic splice donor distal to exon 7 and skipping of exon 7 (Figures 3, B and C). The first form (“MutA”) introduces foreign amino acid codons from intron 7 sequences, causing a premature stop upstream of the IQ motif at the C terminus of the protein (Figures 3, B and C). An exon 7-skipped form (“Mut B”) leaves the reading frame intact but lacking in 54 amino acids. Mutant testes also contain low levels of a normally spliced product, suggesting that the mutation is an extreme hypomorph and not a complete null. The identification of the mutation in *Iqcg* led us to rename the allele *Iqcg^esgd12d^*.

Because *Iqcg^esgd12d^* homozygotes still produce a low level of WT *Iqcg* transcript, this raised two issues. One is whether the mutation in *Iqcg* is really causative of the *esgd12d* phenotype, and the other is whether the *esgd12d* allele and phenotype is hypomorphic. To test these possibilities, we generated mice bearing a mutated allele of *Iqcg* lacking exon 6 (*Iqcg^KO/KO^*) (Figure S1). Because deletion of this 82 bp exon disrupts the reading frame, it would likely constitute a null allele. Western blot analysis revealed a lack of IQCG protein in *Iqcg^KO/KO^* testes, confirming this conclusion (Figure 3D). Testes of homozygous mutants showed histological defects indistinguishable from *Iqcg^esgd12d^* mutants and lacked any motile epididymal sperm (not shown). We then performed complementation analysis of the two alleles. *Iqcg^esgd12d/KO^* males were sterile (N = 2), had no motile epididymal sperm (N = 5), and had histological defects indistinguishable from either single mutant (Figure 1G), thus proving that the T > A mutation in *Iqcg* is responsible for the *esgd12d* phenotype.

**Figure 4:**
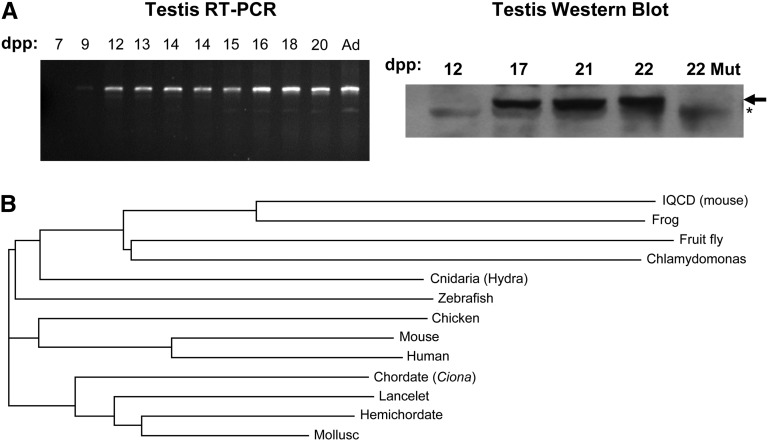
Spermatogenic expression and evolutionary conservation of *Iqcg*. (A) RT-PCR and Western blot analyses of *Iqcg* at the indicated number of days postpartum (dpp). Ad = adult. RNA and protein sources were whole testis. “Mut” = mutant for *Iqcg*. The arrow indicates the IQCG protein band, and the asterisk (*) indicates a nonspecific background species. (B) Evolutionary tree of IQCG protein orthologs plus mouse IQCD (top). The tree was created using ClustalW.

To determine the temporal and developmental pattern of *Iqcg* expression during spermatogenesis, RT-PCR analysis was performed on testis RNA extracted from WT mice at various ages after birth, taking advantage of the synchrony of the first wave of spermatogenesis. As shown in Figure 4A, *Iqcg* transcripts are barely detectable at 9 days postpartum (dpp), at which the most advanced germ cells are leptotene spermatoctyes, but are present abundantly beginning at 12 dpp (corresponding to zygotene spermatocytes) and thereafter. However, IQCG protein was undetectable at 12 dpp; it was present at 17 dpp, when pachytene spermatocytes are present in testes (Figure 4A). Therefore, there is some degree of posttranscriptional regulation.

### IQCG is conserved across diverse organisms

Because there are no other reports concerning the function of *Iqcg*, we performed BLAST searches to identify orthologs in other species for which more functional information might be available. Interestingly, the IQCG protein is highly conserved, having orthologs in diverse species, including vertebrates, Cnidarians, mollusks, primitive chordates (lancelets, tunicates), hemichordates, insects, and unicellular flagellates, but not plants (*Arabidopsis thaliana*) (Figure 4B). The mammalian genome also contains a paralog called *Iqcd*, which also is expressed testis-specifically (Figure 4B) (Su *et al.* 2004). Notably, the *Drosophila melanogaster* ortholog (CG13972) interacts with Calmodulin (CaM) in a yeast two-hybrid assay (Giot *et al.* 2003), and IQ motif-containing proteins are typically associated with CaM regulation (see *Discussion*). An important insight from these analyses is that the *Chlamydomonas* ortholog is present in the flagellum of this species. It was one of 10 IQ motif–containing flagellar proteins found via proteomic screening of this model ciliate (Pazour *et al.* 2005).

## Discussion

IQ motifs are present in several hundred proteins, most notably myosins, but also in a variety of nonmyosin proteins such as neuronal growth proteins, voltage-gated channels, phosphatases, spindle-associated proteins, and sperm surface proteins (Bahler and Rhoads 2002). CaM is a major calcium sensor and orchestrator of regulatory events through its interaction with a diverse group of cellular proteins. Proteins containing IQ motifs are generally associated with Ca^2+^-independent CaM regulation. CaM promotes localized Ca signaling in activities such as myosin contractions, cytoskeletal organization, and mitosis. *Iqcg* is one of several IQ motif–containing genes of unknown function and no other motifs in the mammalian genome (they are labeled *Iqca-k*, with 6 “f” subfamily members). Because CaM activation can stimulate actin cytoskeleton changes, it is possible that the flagellum formation defects in mutants reflect an involvement of IQCG in spermatid morphogenesis. Interestingly, mutations of the IQ motif–containing protein IQCB1 (also known as Nephrocystin-5) cause renal failure and retinitis pigmentosa in humans as a result of ciliary dysfunction (Otto *et al.* 2005).

Ca^2+^ signaling is important for regulating sperm motility, most notably in this context for flagellar function and regulation (Marquez *et al.* 2006; Lindemann *et al.* 1987; Ignotz and Suarez 2005; Ho *et al.* 2002; Darszon *et al.* 2011). However, these roles cannot entirely explain the defects presented in IQCG-deficient mice because spermiogenesis itself is disrupted, as is the integrity of the sperm tail, not just its function. Interestingly, mutation of *Camk4* Ser/Thr kinase (calcium/calmodulin–dependent protein kinase 4), which is present exclusively in the nuclei of elongating spermatids and associates with the chromatin and nuclear matrix, causes a phenotype with some similarities to *Iqcg* knockouts, namely oligoasthenoteratospermia (Wu *et al.* 2000). In *Camk4^−/−^* sperm, the sequential deposition of basic nuclear proteins on chromatin (histones > transition proteins > protamines) is disrupted, with a specific loss of protamine 2 (an *in vivo* substrate of CAMK4) and prolonged retention of transition protein-2 in step-15 spermatids (Wu *et al.* 2000).

Overall, the phenotype of IQCG-deficient mice does not completely resemble that of any reports we were able to find in the literature. More detailed analysis of the sperm anatomical and biochemical defects, including development of a reagent that will permit subcellular localization *in situ*, will be instrumental in elucidating what appears to be a novel mechanistic pathway in mammalian spermiogenesis. Accession numbers used appear in *Materials and Methods*.

## Supplementary Material

Supporting Information

## References

[bib1] Bahler M. Rhoads A., 2002 Calmodulin signaling via the IQ motif. FEBS Lett. 513: 107–113 11911888 10.1016/s0014-5793(01)03239-2

[bib2] Blendy J. A. Kaestner K. H. Weinbauer G. F. Nieschlag E. Schutz G., 1996 Severe impairment of spermatogenesis in mice lacking the CREM gene. Nature 380: 162–165 8600391 10.1038/380162a0

[bib3] Darszon A. Nishigaki T. Beltran C. Trevino C. L., 2011 Calcium channels in the development, maturation, and function of spermatozoa. Physiol. Rev. 91: 1305–1355 22013213 10.1152/physrev.00028.2010

[bib4] Elliott D., 2003 Pathways of post-transcriptional gene regulation in mammalian germ cell development. Cytogenet. Genome Res. 103: 210–216 15051941 10.1159/000076806

[bib5] Eppig J. T. Blake J. A. Bult C. J. Kadin J. A. Richardson J. E., 2012 The Mouse Genome Database (MGD): comprehensive resource for genetics and genomics of the laboratory mouse. Nucleic Acids Res. 40: D881–D886 22075990 10.1093/nar/gkr974 PMC3245042

[bib6] Gan H. Cai T. Lin X. Wu Y. Wang X., 2013 Integrative proteomic and transcriptomic analyses reveal multiple post-transcriptional regulatory mechanisms of mouse spermatogenesis. Mol. Cell. Proteomics 12: 1144–1157. 10.1074/mcp.M112.020123 PMC3650327 23325766

[bib7] Giot L., J. S. Bader, C. Brouwer, A. Chaudhuri, B. Kuang, 2003 A protein interaction map of Drosophila melanogaster. Science 302: 1727–1736 14605208 10.1126/science.1090289

[bib8] Guo X., J. Shen, Z. Xia, R. Zhang, P. Zhang, 2010 Proteomic analysis of proteins involved in spermiogenesis in mouse. J. Proteome Res. 9: 1246–1256 20099899 10.1021/pr900735k

[bib9] Handel M. A. Lessard C. Reinholdt L. Schimenti J. Eppig J. J., 2006 Mutagenesis as an unbiased approach to identify novel contraceptive targets. Mol. Cell. Endocrinol. 250: 201–205 16412559 10.1016/j.mce.2005.12.046

[bib10] Ho H. C. Granish K. A. Suarez S. S., 2002 Hyperactivated motility of bull sperm is triggered at the axoneme by Ca2+ and not cAMP. Dev. Biol. 250: 208–217 12297107 10.1006/dbio.2002.0797

[bib11] Ignotz G. G. Suarez S. S., 2005 Calcium/calmodulin and calmodulin kinase II stimulate hyperactivation in demembranated bovine sperm. Biol. Reprod. 73: 519–526 15878888 10.1095/biolreprod.105.040733

[bib12] Iguchi N. Tobias J. W. Hecht N. B., 2006 Expression profiling reveals meiotic male germ cell mRNAs that are translationally up- and down-regulated. Proc. Natl. Acad. Sci. USA 103: 7712–7717 16682651 10.1073/pnas.0510999103 PMC1472510

[bib13] Lindemann C. B. Goltz J. S. Kanous K. S., 1987 Regulation of activation state and flagellar wave form in epididymal rat sperm: evidence for the involvement of both Ca2+ and cAMP. Cell Motil. Cytoskeleton 8: 324–332 2826020 10.1002/cm.970080405

[bib14] Marquez B. Ignotz G. Suarez S. S., 2006 Contributions of extracellular and intracellular Ca(2+) to regulation of sperm motility: Release of intracellular stores can hyperactivate CatSper1 and CatSper2 null sperm. Dev. Biol. 303: 214–221 17174296 10.1016/j.ydbio.2006.11.007 PMC1885980

[bib15] Nantel F. Monaco L. Foulkes N. S. Masquilier D. LeMeur M., 1996 Spermiogenesis deficiency and germ-cell apoptosis in CREM-mutant mice. Nature 380: 159–162 8600390 10.1038/380159a0

[bib16] Otto E. A., B. Loeys, H. Khanna, J. Hellemans, R. Sudbrak, 2005 Nephrocystin-5, a ciliary IQ domain protein, is mutated in Senior-Loken syndrome and interacts with RPGR and calmodulin. Nat. Genet. 37: 282–288 15723066 10.1038/ng1520

[bib17] Pazour G. J. Agrin N. Leszyk J. Witman G. B., 2005 Proteomic analysis of a eukaryotic cilium. J. Cell Biol. 170: 103–113 15998802 10.1083/jcb.200504008 PMC2171396

[bib18] Sadate-Ngatchou P. I. Payne C. J. Dearth A. T. Braun R. E., 2008 Cre recombinase activity specific to postnatal, premeiotic male germ cells in transgenic mice. Genesis 46: 738–742 18850594 10.1002/dvg.20437 PMC2837914

[bib19] Su A. I., T. Wiltshire, S. Batalov, H. Lapp, K. A. Ching, 2004 A gene atlas of the mouse and human protein-encoding transcriptomes. Proc. Natl. Acad. Sci. USA 101: 6062–6067 15075390 10.1073/pnas.0400782101 PMC395923

[bib20] Tsai-Morris C. H. Sheng Y. Lee E. Lei K. J. Dufau M. L., 2004 Gonadotropin-regulated testicular RNA helicase (GRTH/Ddx25) is essential for spermatid development and completion of spermatogenesis. Proc. Natl. Acad. Sci. USA 101: 6373–6378 15096601 10.1073/pnas.0401855101 PMC404052

[bib21] Ward J. O., L. G. Reinholdt, S. A. Hartford, L. A. Wilson, R. J. Munroe, 2003 Toward the genetics of mammalian reproduction: induction and mapping of gametogenesis mutants in mice. Biol. Reprod. 69: 1615–1625 12855593 10.1095/biolreprod.103.019877

[bib22] Wu J. Y. Ribar T. J. Cummings D. E. Burton K. A. McKnight G. S., 2000 Spermiogenesis and exchange of basic nuclear proteins are impaired in male germ cells lacking Camk4. Nat. Genet. 25: 448–452 10932193 10.1038/78153

[bib23] Zhang D. Penttila T. L. Morris P. L. Teichmann M. Roeder R. G., 2001 Spermiogenesis deficiency in mice lacking the Trf2 gene. Science 292: 1153–1155 11352070 10.1126/science.1059188

