# Supplementary material for: IQ Motif-Containing G (Iqcg) Is Required for Mouse Spermiogenesis
Source: G3 (Bethesda). 2013 Dec 20;4(2):367–72. doi: 10.1534/g3.113.009563 (PMC3931569; doi:10.1534/g3.113.009563)
Supplement: Supporting Information [file supp_4_2_367__index.html]

IQ Motif-Containing G (Iqcg) Is Required for Mouse Spermiogenesis — Supporting Information 

# IQ Motif-Containing G (*Iqcg*) Is Required for Mouse Spermiogenesis

## Supporting Information for Harris *et al.*, 2014

**Files in this Data Supplement:**

- Supporting Information - Figure S1 and Table S1 (PDF, 472 KB)
- Figure S1 - Structure of *Iqcg* null allele. (PDF, 332 KB)
- Table S1 - Recombinant chromosomes in the vicinity of esgd12d (Iqcg) on Chr 16. (.xls, 352 KB)
