# Supplementary material for: IQ Motif-Containing G (Iqcg) Is Required for Mouse Spermiogenesis
Source: G3 (Bethesda). 2013 Dec 20;4(2):367–72. doi: 10.1534/g3.113.009563 (PMC3931569; doi:10.1534/g3.113.009563)
Supplement: Supporting Information [file supp_g3.113.009563_009563SI.pdf]

G3: Genes | Genomes | Genetics

IQ motif-containing G (*Iqcg*) is required for mouse spermiogenesis

Tanya P. Harris*, Kerry J. Schimenti, Robert J. Munroe and John C. Schimenti

Dept. of Biomedical Sciences, and the Dept. of Molecular Biology and Genetics, Cornell University, Ithaca, NY 14853

* Current address: Fertility Associates, Christchurch, NZ.

Corresponding author:
John Schimenti, PhD
Cornell University
College of Veterinary Medicine T9014A
Ithaca, NY 14853
TEL: 607-253-3636
jcs92@cornell.edu

DOI: 10.1534/g3.113.009563

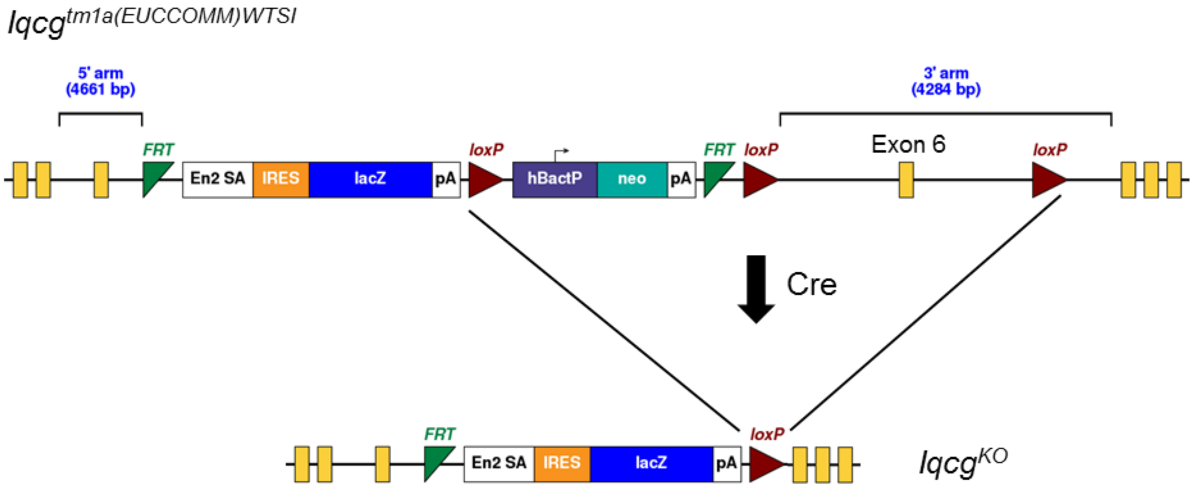

Figure S1 Structure of *Iqcg* null allele. The targeted allele at the top was generated at The Sanger Institute, and this is a screen shot from the KOMP (Knockout mouse project) web site (http://www.knockoutmouse.org/martsearch/project/36432). See methods for details.

Table S1 Shown are recombinant chromosomes in the vicinity of esgd12d (Iqcg) on Chr 16. These came from crosses involving C3H or CAST as the WT partner ("C"); the esgd12d mutation arose on C57BL/6J ("B"). Many of the informative recombinants required progeny testing (because either the mutation was in trans to C, or the recombinant was a female). Multiple males arise from a progeny test were grouped in shaded and unshaded rows. The esgd12d critical region indicated in green. Markers used for mapping include microsatellite markers abbreviated with an M for "D16Mit". E4 is a custom marker (see Methods). Mb positions are from assembly mm10).

Table S1 is available for download as an Excel file at http://www.g3journal.org/lookup/suppl/doi:10.1534/g3.113.009563/-/DC1
