# Supplementary material for: IQ Motif-Containing G (Iqcg) Is Required for Mouse Spermiogenesis
Source: G3 (Bethesda). 2013 Dec 20;4(2):367–72. doi: 10.1534/g3.113.009563 (PMC3931569; doi:10.1534/g3.113.009563)
Supplement: Supporting Information [file supp_g3.113.009563_FigureS1.pdf]

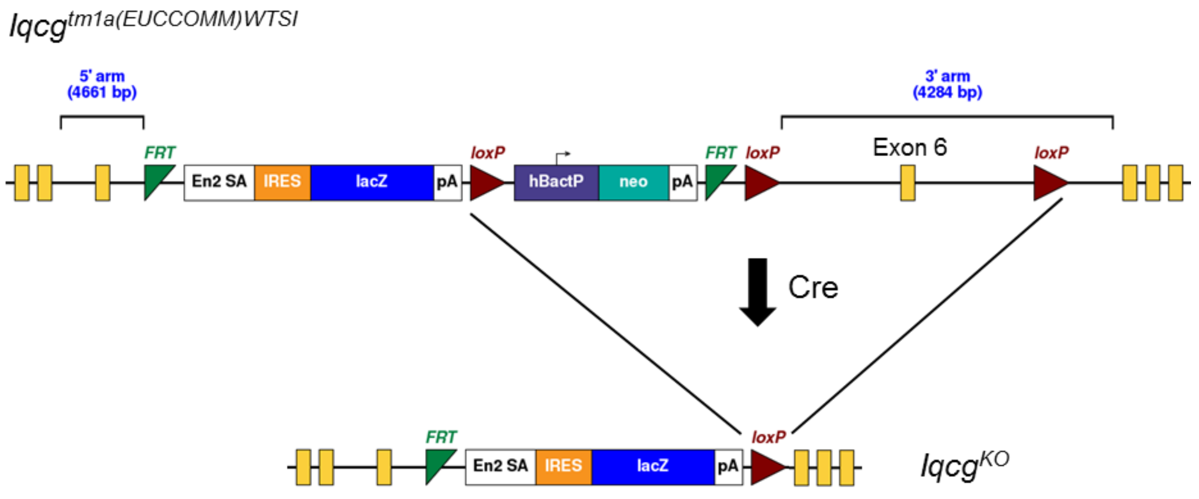

Figure S1 Structure of *Iqcg* null allele. The targeted allele at the top was generated at The Sanger Institute, and this is a screen shot from the KOMP (Knockout mouse project) web site (http://www.knockoutmouse.org/martsearch/project/36432). See methods for details.
